# Genetics of digital phenotypes of keel bone in layer chickens and correlations with keel bone fractures and deviations

**DOI:** 10.1186/s12711-025-01016-7

**Published:** 2025-11-27

**Authors:** Moh Sallam, Lina Göransson, Anne Larsen, Helena Wall, Wael Alhamid, Stefan Gunnarsson, Martin Johnsson, Dirk-Jan de Koning

**Affiliations:** 1 Department of Animal Biosciences, Swedish University of Agricultural Sciences (SLU), Box 7023, S-750 07 Uppsala, Sweden; 2 Department of Applied Animal Science and Welfare, Swedish University of Agricultural Sciences (SLU), 532 23 Skara, Sweden Box 234,

## Abstract

**Background:**

Poultry is a global industry with laying hens that are genetically optimized for high egg yield. Keel bone fractures can affect up to 80% of laying hens, posing welfare and production problems. Therefore, genetic selection to reduce keel fractures is important. However, the lack of a reliable, automated, and heritable phenotypes for keel bones makes this a challenging task. The aim of this study was to (1) develop automated analyses of radiographic images to phenotype keel bones, and (2) investigate whether the proposed phenotypes are heritable and genetically correlated with the post-dissection scores of keel bone fractures and deviations. A total of 1051 laying hens (Bovans Brown and Lohmann Brown) from a commercial farm were x-rayed, followed by keel bone dissection and scoring for deviations and fractures. Furthermore, blood was sampled for genotyping using 50 K Illumina SNP chips. Keel bones were segmented (with ~ 0.90 accuracy) from the radiographic images using deep learning models, after which the images were automatically measured for general geometry and radiopacity. Multi-trait genomic restricted maximum likelihood was used to estimate genetic parameters.

**Results:**

Heritability estimates ranged from 0.28 to 0.30 for both keel deviations and fractures observed post-dissection. The automated phenotypes had heritability estimates ranging from 0.07 to 0.10 for keel radiopacity and from 0.11 to 0.39 for keel geometry. Estimates of genetic correlations of keel geometry with keel deviation and fractures ranged from -0.57 to 0.72.

**Conclusions:**

Automated methods were developed for measuring keel bone radiopacity and geometry. Keel concave area was found to be a reliable and heritable phenotype that breeding companies can use to reduce keel deviations and fractures. These methods can also be adapted to measure other bones (e.g., tibiotarsal) or objects (e.g., eggs), allowing breeders to quickly compute phenotypes for keel, tibia, and egg size from the same radiographic image. The developed methods are well-suited for large-scale studies to assess different housing environments and nutrition strategies aimed at improving keel bone conditions.

**Supplementary Information:**

The online version contains supplementary material available at 10.1186/s12711-025-01016-7.

## Background

Poultry production is a global industry, where laying hens are genetically optimized for high egg yield. However, despite these advancements, keel bone damage, including fractures and other non-fracture related deformities (deviations), remains a prevalent issue, affecting up to 80% of laying hens [1–5], posing welfare [6, 7] and production [8] problems. Bone health in laying hens is a multifactorial issue, shaped by (1) genetic or animal-based traits such as age at first egg and egg size, (2) environmental conditions like housing systems and nutrition, and (3) potential genotype-by-environment interactions [9–12]. Although poultry nutrition and housing have notably advanced [12–15], keel bone damage remains common. The heritability of bone traits is generally well-established in laying hens [16–18], suggesting that genetic improvement is theoretically achievable. The poultry industry therefore seeks to reduce keel bone damage through genetic selection, while maintaining production levels. The crucial question is how poultry breeding companies can assess the keel bones of thousands of birds to provide meaningful phenotypes that can drive genetic selection to improve keel bone health.

Current methods for examining bones of chickens are invasive, such as post-mortem bone dissections, and non-invasive, such as palpation and radiographic imaging (X-ray) of live birds. In the dissection techniques, the bones are separated from the adjacent tissues, so that scoring bones post-dissection is very reliable, and usually used as the ground truth to validate the non-invasive techniques, which are preferred for selective breeding methods, especially if performed on live birds.

Palpation of live birds by skilled assessors is the simplest non-invasive method to score keel bones [19–22] but has some limitations. Unless the keel fractures are large enough to form substantial calluses, palpation methods tend to underestimate their true prevalence [19, 23–26]. In non-cage systems, where birds can move freely, keel fractures often form severe pronounced calluses [27], making palpation more effective in such systems. However, as a human-based assessment, palpation is inherently subjective, prone to assessor bias, and requires extensive training for reliable outcomes [28]. At best, palpation outcomes are either binary (fractured vs. non-fractured) or on a severity scale from 1 to 4 [19–22], neither of which offers fully continuous data.

Radiography provides non-invasive imaging of bones and has been proposed as a potential solution to evaluate keel bones of live birds [29–32]. The use of a simple restraint tool can minimize the birds’ discomfort during radiographic examinations [32] and can be applied under farm conditions [31]. Radiography produces a two-dimensional image of the keel bone, but with potential noise from the adjacent tissues. Therefore, the radiographic findings must be compared to post-dissection findings to investigate their agreement. For instance, detection of keel bone damage on X-ray images agreed with the post-dissection findings in 85% of keel fractures but in only 60% of keel deviations [24].

Current methods using radiography require skilled human-operators to score keel fractures and deviations on the images [25, 29, 30, 33, 34]. Operators can also mark key points on radiographic images to calculate bone radiopacity and geometry [31, 32, 34]. Human processing of radiographic images is, however, time and labour consuming, as well as prone to noise and bias, and is not a feasible approach for evaluating thousands of birds in a poultry breeding operation. Instead, an automated method is needed to efficiently use radiographic images for phenotyping for the purpose of selective breeding.

Fully automated processing of radiographic images, with validated outcomes on a continuous rather than a binary scale, is expected to provide better keel bone phenotypes than manual palpation of live birds. If these phenotypes are accurate and heritable, they can guide breeders in selecting birds with a lower risk of keel fractures and deviations. If keel bone phenotypes vary mainly due to non-genetic factors, they can help identify management practices such as housing and nutrition to reduce keel fractures and deviations.

The objectives of the present work were to (1) develop an automated method for phenotyping keel bones on radiographic images, and (2) determine whether the developed phenotypes are heritable and genetically correlated with post-dissection scores of keel bone fractures and deviations.

## Methods

### Birds, housing and management

Laying hens of brown hybrids (Bovans Brown and Lohmann Brown) from six flocks on a commercial farm in Sweden were included in the study. Each flock of 5500 birds was separately housed, and the barns were depopulated in April (2021, 2022), August (2021, 2022), and December (2021, 2022), respectively. The choice of hybrid type: Bovans (4 flocks) vs. Lohmann (2 flocks) was made by the farmer. All birds were kept in aviaries under similar lighting and feeding conditions, and at a stocking density of 9 hens per m² available area. The birds (non-beak trimmed) arrived at 16 weeks of age and the barns were depopulated around 73 to 76 weeks of age.

Several days prior to depopulation, the birds evaluated in this study were opportunistically sampled (~ 120 birds from each of the first five flocks and 500 birds from the last flock). Due to logistic constrains, it was not possible to sample more birds from the first flocks. The last flock also included a longitudinal study of around 250 birds that were radiographed multiple times [31]. The sampled birds were individually weighed using a digital scale and clinically scored for keel bone damage using palpation, plumage condition, and foot health. Immediately after scoring, the bird was stunned with a hard blow to the head, killed by neck dislocation, and exsanguinated. A blood sample was then collected for DNA extraction and genotyping. Each bird was marked, packed in a plastic bag, and frozen (−20 °C) at the Götala Beef and Lamb Research Centre of the Swedish University of Agricultural Sciences, Sweden. All sampled birds (*n* = 1150) were then analysed further during two weeks of dissection and post-mortem analyses. The thawed birds were measured for pelvic capacity (see [31] for details) and underwent whole-body radiography, followed by dissection of the keel and tibiotarsal bones. The dissected bones were then radiographed as described in in [31], followed by post-dissection keel bone scoring.

## Radiographic keel bone measurements

**Fig. 1:**
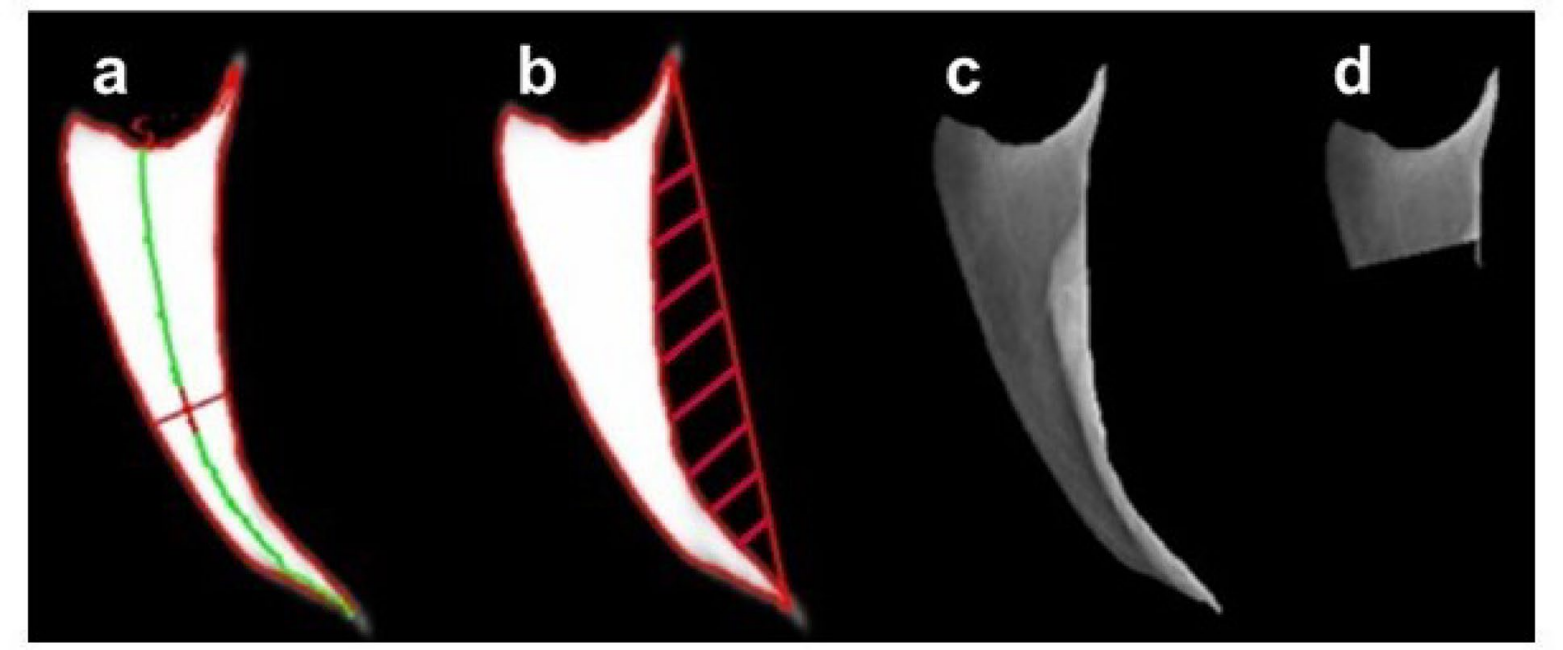
Keel bone phenotypes Legend: (**a**) the ratio of keel length (green line) to mid-depth (red line intersects with keel contour at two points); (**b**) the concave area (red shading) at the keel visceral or dorsal side; radiopacity is the average of pixels’ intensities — (**c**) across the whole keel bone or (**d**) across the keel cranial fifth. keel bone orientations: cranial at the top, caudal at the bottom, ventral to the left, and dorsal to the right

Radiographic imaging was performed using a portable X-ray machine. Birds were positioned over the detector panel at a fixed distance of 100 cm from the X-ray source, which was set to 60 kilovolts and 1.6 milliampere-seconds per exposure, as described and illustrated in detail in our earlier work [31]. Keel bones were segmented (with ~ 0.90 accuracy) from the radiography images using a deep learning algorithm, as described in [35]. The segmented keel bones were then automatically measured for (a) keel bone length and mid-depth, (b) keel concave area, radiopacity of (c) whole keel and (d) keel cranial fifth (more detail below). The algorithms to automate these measurements were developed using the Python language and the open-source computer vision library OpenCV (www.opencv.org), with an example of outcomes shown in Fig. 1.

The segmentation algorithm processes whole-body radiography images, returning images of the same dimensions but with the keel bone highlighted in gray-white and the remainder of the X-ray image as black background [35]. The contrast between keel bone and the background is therefore large enough to determine the contour of the keel bone. The contour is a list of points that outline the keel bone, with each point given as (x, y) coordinates in 2-dimensional space. We used several operations on the keel bone contour to automate the following measurements:

1. Averaging x coordinates over the points with the same y coordinate, result in the green line in Fig. 1a, corresponding to the keel bone length.
2. Drawing a line that passes vertical at the mid-point of the green line while intersecting with the keel contour resulted in the red straight line in Fig. 1a, corresponding to the keel bone mid-depth.
3. In computer vision, the origin point of the coordinate system (x = 0, y = 0) is typically located at the most upper-left corner of the image. The longest straight red line in Fig. 1b is drawn between the points of (maximum x, minimum y) and (maximum x, minimum y) of the keel bone contour, and the difference between the keel bone area after and before drawing this line is the concave area (red shading in Fig. 1b). Based on this, we defined keel concave area as the area of the concavity on the dorsal (visceral) side of the keel bone. This is typically the side of the keel that faces the internal organs.
4. Cropping the radiographed whole-body image for the whole keel bone contour resulted in Fig. 1c, while cropping for the cranial (upper) fifth of the keel bone contour resulted in Fig. 1d. The average pixel intensity of the cropped images is referred to as bone radiographic density or radiopacity, which is the term we will use the remainder. Bone radiopacity refers to the degree to which bone appears opaque or dense on the X-ray images and indicates the amount of X-ray absorption by the bone. Denser bones, which contain more minerals, absorb more X-rays and thus appear whiter or more opaque on X-ray images. Conversely, less dense bones appear darker.

## Keel bone scoring post-dissection

Nine technicians and researchers, trained by two veterinarians, scored the dissected keel bones that were randomly assigned to them, with each operator scoring at least 85 bones. Keel bones were scored for deviation size (3 categories: none, < 0.5 cm, and ≥ 0.5 cm), number of fractures (5 categories: none, 1, 2, 3, ≥ 4), and callus size (3 categories: none, mild to moderate, and severe). The details of the keel bone scoring protocol can be found in [31].

## Other traits evaluated

Body weight, pelvic capacity, and radiopacity of tibiotarsal bone post-dissection were also evaluated. This was to estimate their genetic correlation with the keel bone traits. These traits were measured manually by one technician for trait. Pelvic capacity was obtained as pelvic width times pelvic depth, as described in [31]. Radiopacity was measured at the mid-shaft of tibiotarsal bone following the method of Wilson et al. [32] and as applied in [31].

## Comparing average radiography phenotypes across levels of keel bone damage

The proposed radiography phenotypes are on a continuous scale, while the keel bone damage traits are on ordinal categorical scale. To investigate whether the radiographic phenotypes are relevant to keel bone damage, average radiographic measurements of different parts of the keel bone were compared across the levels of damage scored on keel bones post-dissection, using Tukey’s all-pairwise comparisons, as implemented in the R package “stats” [36].

### Genotypes

The DNA was extracted from the blood samples and all hens were genotyped for 57,636 single nucleotide polymorphisms using the Illumina Infinium assay [37, 38]by the SNP&SEQ Technology Platform, Uppsala University, Sweden. A total of 17,619 SNPs were removed because of being monomorphic or having a low SNP call rate (< 0.90) or minor allele frequency (< 0.05). After all quality control checks, a total of 40,017 SNPs markers were retained for further analysis.

## Principal component analysis

Principal component analysis was used to cluster individuals with similar SNP genotypes. Genotypes of birds from the two hybrids and flocks were combined and analyzed using the “pca” function in the PLINK software [39]. The resulting files of eigenvalues and eigenvectors where then visualized using the R package “ggplot2” [40].

## Estimation of genetic parameters

For estimation of genetic parameters, we used a nine-trait genomic animal model:

$$\begin{gathered} \left[ {\begin{array}{*{20}c} {{\mathbf{y}}_{1} } \\ {{\mathbf{y}}_{2} } \\ \vdots \\ {{\mathbf{y}}_{9} } \\ \end{array} } \right] = \left[ {\begin{array}{*{20}c} {{\mathbf{X}}_{1} } & 0 & \cdots & 0 \\ 0 & {{\mathbf{X}}_{2} } & \cdots & 0 \\ \vdots & \vdots & \ddots & \vdots \\ 0 & 0 & \cdots & {{\mathbf{X}}_{9} } \\ \end{array} } \right]\left[ {\begin{array}{*{20}c} {{\mathbf{b}}_{1} } \\ {{\mathbf{b}}_{2} } \\ \vdots \\ {{\mathbf{b}}_{9} } \\ \end{array} } \right] \hfill \\ + \left[ {\begin{array}{*{20}c} {{\mathbf{Z}}_{1} } & 0 & \cdots & 0 \\ 0 & {{\mathbf{Z}}_{2} } & \cdots & 0 \\ \vdots & \vdots & \ddots & \vdots \\ 0 & 0 & \cdots & {{\mathbf{Z}}_{9} } \\ \end{array} } \right]\left[ {\begin{array}{*{20}c} {{\mathbf{u}}_{1} } \\ {{\mathbf{u}}_{2} } \\ \vdots \\ {{\mathbf{u}}_{9} } \\ \end{array} } \right] + \left[ {\begin{array}{*{20}c} {{\mathbf{e}}_{1} } \\ {{\mathbf{e}}_{2} } \\ \vdots \\ {{\mathbf{e}}_{9} } \\ \end{array} } \right], \hfill \\ \end{gathered}$$,

where **y** is a vector of phenotypic values for each trait, with subscripts 1 to 9 denoted the following traits : 1 and 2 = deviation size and fracture count obtained post dissection ; 3 = pelvic capacity; 4 = slaughter body weight; 5 and 6 = keel bone concave area and ratio of length to mid-depth; 7 to 9 = radiopacity of whole keel bone, keel cranial fifth, and tibiotarsal mid-shaft. **X** is a design matrix that relates **y** to the vector **b** of the fixed effects, which included hatch (*n* = 6), house (*n* = 4), hatch by house (*n* = 6), and cluster membership (*n* = 2) from the principal component analysis. **Z **is a design matrix that relates y to the vector **u **of random hen additive genetic effects.

The hen (u) and residual (e) effects were assumed to follow a multivariate normal distribution:

$$\:\mathbf{u}\sim\:N\left(0,\mathbf{G}\otimes\:{\varvec{\Sigma\:}}_{\mathbf{u}}\right)$$

$$\:\mathbf{e}\sim\:N\left(0,{\varvec{\Sigma\:}}_{\mathbf{e}}\right)$$

where $\:\mathbf{G}$ is the genomic relationship matrix among animals. $\:{\varvec{\Sigma\:}}_{\mathbf{u}}$ and $\:{\varvec{\Sigma\:}}_{\mathbf{e}}$ are the variance-covariance matrix of the genetic and residual effects across traits. The genomic relationship matrix** G** was constructed using first method of VanRaden [41]:

$$\:\mathbf{G}=\:\frac{\mathbf{W}\:{\mathbf{W}}^{{\prime\:}}\:}{\sum\:2{p}_{i}\:\left(1-{p}_{i}\right)}$$,

where **W** is a matrix of n genotyped individuals × m SNPs and contains SNP genotypes coded as 0, 1, and 2 for major allele homozygous, heterozygous, and minor allele homozygous, respectively. Each column of **W** represents genotypes per individuals for one SNP, centred by subtracting the minor allele frequency *p* from its elements.

Prior to analysis, phenotypic values were standardized per trait by subtracting the mean and dividing by the standard deviation. This resulted in **y** having a mean of zero and a standard deviation of one, facilitating numerical computation, model convergence, and interpretation across traits. All traits included in the analysis were treated as continuous. To account for differences in scoring between technicians (*n* = 9), keel bone deviation size and fracture count were pre-adjusted by taking the deviation of each trait score from the average score for that technician.

Genetic parameters (heritability and genetic correlations) and their standard errors were estimated using the multi-trait genomic restricted maximum likelihood (GREML) via the average information algorithm, as implemented in the AIREMLF90 package [42, 43].

## Results

We examined the keel bones of 1006 birds post-dissection. Damage was found in 94% of the keel bones examined. The damage comprised deviations (81%), fractures (85%), and/or calluses (81%). The latter two had a high co-frequency of 81%. The co-frequency of deviations and fractures was 72%. Thus, some fractures (13%) and deviations (9%) occurred independently of each other.

### Average radiography phenotypes across levels of keel bone damage

**Fig. 2:**
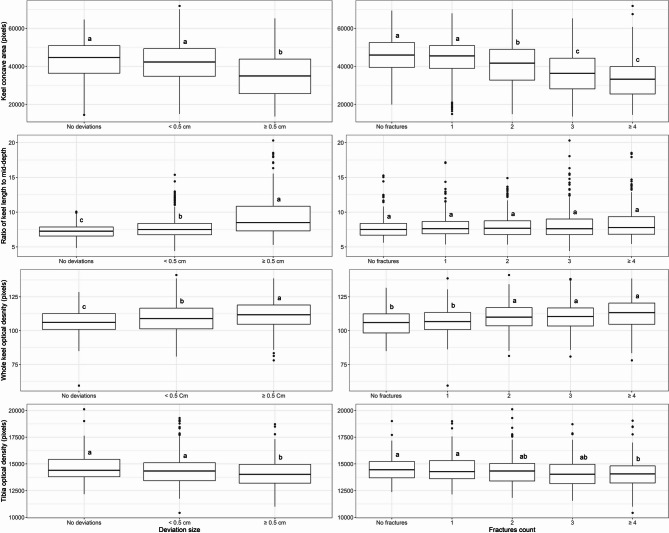
Average radiography phenotypes by levels of keel bone deviation and fracture count Legend:. Different letters on score group boxes indicate significantly different average value (Tukey statistics, *p* < 0.05)

Averages of radiography phenotypes by level of keel bone damage, as evaluated post-dissection, are shown in Fig. 2. Keel bone concave area was on average larger in keels with no or slight deviations compared to keels with severe deviations. The concave area was on average also larger in keels with no or one fracture, moderate in birds with two fractures, and smaller in keels with three or more fractures (Fig. 2 first row). The ratio of keel bone length to mid-depth was on average small in keels with no deviations, moderate in keels with slight deviations, and high in keels with severe deviations. However, the keel bone length-to-depth ratio was on average not different between levels of keel bone fractures (Fig. 2 second row).

Birds with severe keel bone damage on average had lower radiopacity in tibia, but higher radiopacity in the whole keel, compared to birds with damage-free keels (Fig. 2 third and fourth row). Whole keel bone radiopacity was on average small, moderate, and high in keels with, respectively, no, slight, and severe deviations. Whole keel bone radiopacity was on average also lower in keels with no or one fracture than in keels with two or more fractures. Radiopacity of the cranial fifth of keel bone was on average not different between levels of keel bone deviations or fractures [See Additional file 1, Fig. S1].

### Estimates of genetic parameters

**Fig. 3:**
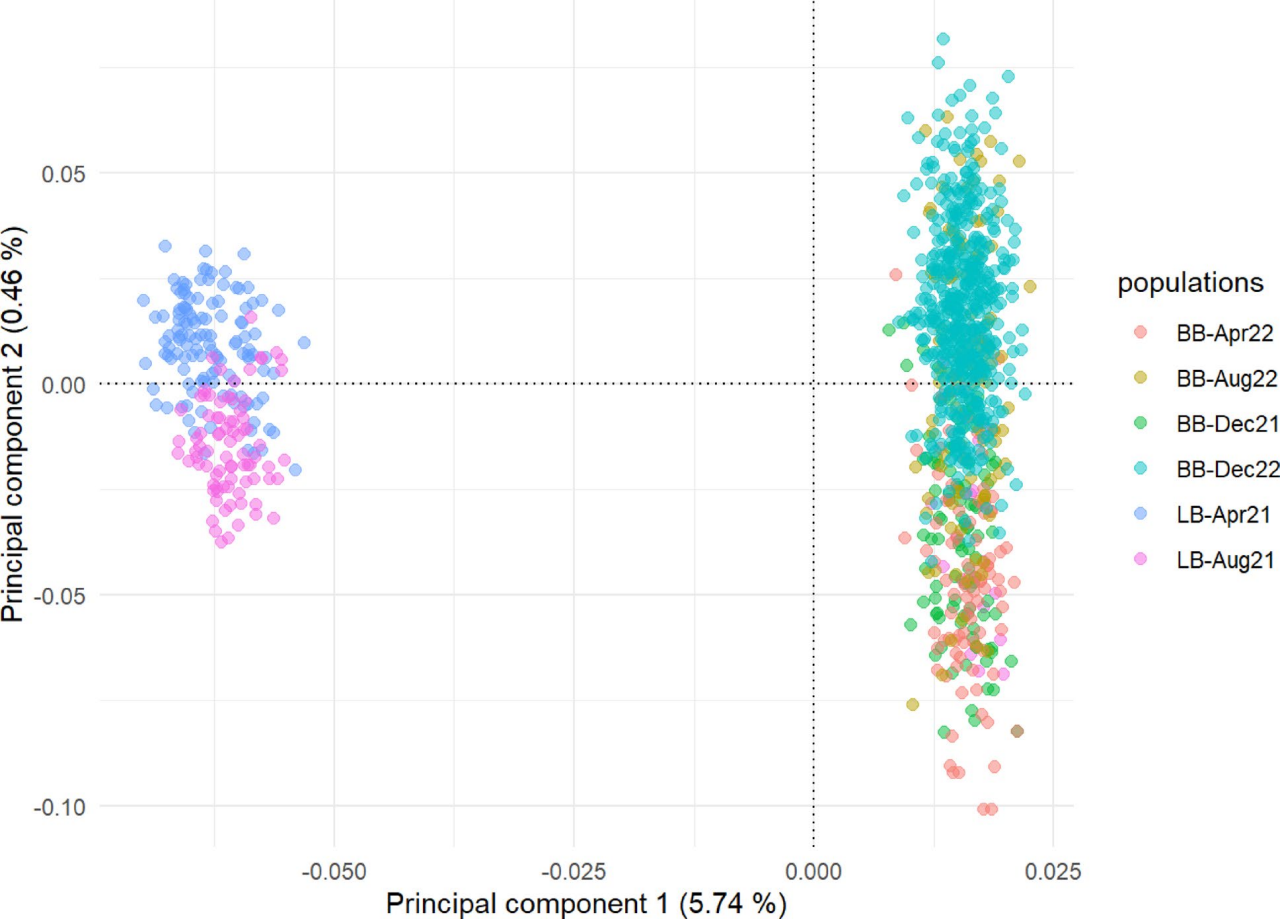
Principal component analysis of genotypes of birds of the two hybrids (LB: Lohmann Brown and BB: Bovans Brown) from six batches

Principal component analysis of the genotypes identified two clusters (Fig. 3): one cluster with hybrids of Bovans Brown from different batches (770 birds) and one cluster of hybrids of Lohmann Brown (217 birds).

The genetic parameters were estimated using data of all birds (*n* = 987), with hybrid type included as a fixed effect, in Table 1. The genetic parameters were also estimated using only data of Bovans hybrids (*n* = 770) [See Additional file 2, Table S1]. Using full or partial data resulted in similar estimates of genetic parameters; results from the full data combining the two hybrids are reported below. Estimates of heritability based on single trait GREML are reported in Additional file 3, Table S2.

**Table 1:** Estimates ± standard error of heritabilities (diagonals and bolded), genetic correlations (lower off-diagonals), and phenotypic correlations (upper off-diagonals) of traits evaluated on Bovans brown and Lohmann brown birds

	Keel bone deviations size^a^	Keel bone fractures count^a^	Pelvic capacity^b^	Culling body weight	Keel bone concave area^c^	Ratio of keel bone length to mid-depth^c^	Whole keel bone radiopacity^c^	Tibiotarsal bone radiopacity^d^	Keel bone cranial fifth radiopacity^c^
Keel bone deviations size	0.28 ± 0.08	0.31 ± 0.03	0.11 ± 0.04	−0.01 ± 0.04	−0.28 ± 0.03	0.35 ± 0.02	0.21 ± 0.03	−0.11 ± 0.04	0.08 ± 0.03
Keel bone fractures count	0.70 ± 0.20	0.30 ± 0.06	0.11 ± 0.04	−0.05 ± 0.04	−0.39 ± 0.03	0.11 ± 0.03	0.20 ± 0.03	−0.15 ± 0.03	0.08 ± 0.03
Pelvic capacity	0.11 ± 0.21	0.06 ± 0.17	0.33 ± 0.06	0.48 ± 0.03	−0.41 ± 0.03	0.02 ± 0.03	0.02 ± 0.03	0.06 ± 0.04	−0.02 ± 0.03
Culling body weight	−0.08 ± 0.23	0.03 ± 0.15	0.52 ± 0.14	0.44 ± 0.08	−0.11 ± 0.03	−0.01 ± 0.03	−0.08 ± 0.03	0.35 ± 0.03	−0.07 ± 0.04
Keel bone concave area	−0.57 ± 0.21	−0.57 ± 0.13	−0.54 ± 0.14	−0.36 ± 0.17	0.39 ± 0.07	−0.07 ± 0.03	−0.31 ± 0.03	0.10 ± 0.04	−0.14 ± 0.03
Ratio of keel bone length to mid–depth	0.63 ± 0.33	0.72 ± 0.31	−0.03 ± 0.32	−0.14 ± 0.29	−0.31 ± 0.32	0.11 ± 0.05	0.26 ± 0.03	−0.06 ± 0.03	0.06 ± 0.03
Whole keel bone radiopacity	0.77 ± 0.29	0.61 ± 0.40	−0.15 ± 0.25	−0.03 ± 0.27	−0.60 ± 0.29	0.53 ± 0.38	0.10 ± 0.03	−0.10 ± 0.03	0.89 ± 0.07
Tibiotarsal bone radiopacity	−0.25 ± 0.20	−0.37 ± 0.13	−0.04 ± 0.16	0.33 ± 0.14	0.17 ± 0.15	0.03 ± 0.27	−0.41 ± 0.24	0.52 ± 0.08	−0.08 ± 0.03
Keel bone cranial fifth radiopacity	0.40 ± 0.76	0.22 ± 0.94	−0.21 ± 0.54	0.11 ± 0.52	−0.31 ± 0.49	0.31 ± 1.43	0.84 ± 0.67	−0.32 ± 0.70	0.07 ± 0.05

^a^ scoring keel bones post-dissection by nine operators, then adjusting the scores for the operator effect

^b^ requires human-operator to measure pelvic width and depth by digital calliper

^c^ fully automated measurement on the radiographs of chicken whole-body

^d^ requires human-operator to indicate key points on radiographs of the dissected tibiotarsal bones, from these points computes the tibiotarsal bone mid-shaft radiopacity

For the keel bone geometry, concave area had higher estimate of heritability than the ratio of length to mid-depth (0.39 versus 0.11, Table 1). The latter showed slightly stronger genetic correlation estimates with deviations and fractures, than the concave area did (0.72 versus 0.57 for fractures; 0.63 versus 0.57 for deviations). Keel bone concave area showed genetic correlation estimates of  −0.41 with pelvic capacity and − 0.60 with whole keel bone radiopacity, Table 1.

Radiopacity of tibiotarsal bone had higher heritability estimate than whole and cranial fifth of keel bone (0.52 versus 0.10 and 0.07 respectively) (Table 1). Keel bone deviations and fractures had weak favourable genetic correlation estimates (−0.25 and −0.37) with tibiotarsal bone radiopacity, and strong unfavourable genetic correlation estimates (0.77 and 0.61) with whole keel bone radiopacity. Radiopacity of keel bone cranial fifth had very low heritability and noisy genetic correlation estimates.

## Discussion

In the current work, we developed and evaluated radiographic phenotypes of keel bones. This novel method allows the keel bone to be automatically segmented and measured for geometry and radiopacity using radiographic images. The radiographic measurements and the post-dissection scores of keel bones on the same birds were analysed simultaneously with other traits, for estimation of heritability and genetic correlations.

The heritability estimates of ~ 0.30 for both keel bone deviations and fractures (post-dissection scores) suggest the possibility of their used for genetic improvement of keel bone health. However, the dissection itself is too time and labour consuming to efficiently phenotype keel bones. Also, genetic selection based on the post-dissection phenotypes may not be efficient, as the phenotyped individuals must be relatives of the selection candidates. To enable poultry breeders to select for keel bone traits without dissecting keel bones, we developed non-invasive phenotypes/assessments of keel bone using radiography. While radiography as a method for assessing birds’ keel bones is not new [24, 26, 29, 30, 33, 34, 44], the current work introduces novel methods for automatically measuring keel bones in the radiographic images: based on the automated dissection of the keel bone from the whole-body X-ray in [35], we added here automated measurements of the different keel bone dimensions on the dissected image. Rather than manually scoring the radiographic images for keel bone deviation and fractures, we developed automated continuous phenotypes that show moderate heritability estimates and good estimates of genetic correlations with the dissection traits. This automation is valuable as it enables poultry breeders to compute keel bone phenotypes from thousands of images instantly, facilitating selective breeding. It can also enable the evaluation of different housing and nutrition strategies that aim to improve keel bone conditions.

The genetic parameters estimated in the current study pertain to hybrids and may, therefore, reflect genetic variation within and between the pure breeding lines. Estimation of these parameters in pure lines, and their genetic correlations in the hybrids are needed for their application to selection within pure lines. Heterosis may also contribute to the observed heritability, but this cannot be quantified without data from the pure lines.

### Radiographic keel bone geometry

While all proposed phenotypes (i.e., keel concave area, keel length etc.) can be automatically measured, they vary in estimates of heritability and of genetic correlations with keel bone damage traits and, therefore, in their usefulness as indicator traits to select for keel bone health. In this study, keel bone concave area appears to be the most useful keel bone phenotype because it (1) has a higher heritability estimate than other keel bone phenotypes and (2) has moderate to high phenotypic and genetic correlations with both keel bone deviations and fractures, i.e., the postmortem damage parameters that selection would aim to decrease. Thus, when evaluating keel bone concave area, there is no need to assess keel bone deviation and fractures separately [25, 29], areas it can be used as a proxy for both, i.e. the larger the keel bone concave area the lower the keel bone deviations and fractures.

Previously, we found that the ratio of keel bone length to mid-depth is relevant to keel bone damage [31]. However, its heritability estimate was too low (0.11–0.13) for its use as an indicator trait to select for decreased incidence of keel bone fractures. However, it has moderate correlations with keel bone deviations, and is easy to measure with radiography, using the protocols described here.

### Radiographic keel bone radiopacity

Both whole and cranial keel bone radiopacity had heritability estimates ranging from 0.07 to 0.10, in line with Dunn et al. [16]. The current finding of a positive and unfavourable genetic correlation estimate of keel bone radiopacity with keel bone damage is, however, quite problematic since it implies that high radiopacity can be the result of stronger bones or more callus after fractures. These findings contrast with the study of Bishop et al. [44], who reported radiographic keel bone radiopacity (density) to have moderate heritability and a negative (favourable) genetic correlation with bone fractures. However, the current study was conducted in aviary-housed birds, while birds in the Bishop et al. [44] were housed in a non-furnished cage system and bone radiopacity/strength is known to be influenced by housing conditions [10, 45]. The greater variability between different non-cage systems (multi-tiers, perches, etc.) compared to cages may explain differences in heritability of keel bone radiopacity in our study compared to the cage-housed birds in Bishop et al. [44].

Our heritability estimate of tibiotarsal bone radiopacity was ~ 0.5, in line with estimates in [16, 46]. Our estimate of the genetic correlation between tibiotarsal bone radiopacity and keel bone damage was favourable, but not that strong (−0.25 to −0.37). Tibiotarsal bone radiopacity and keel bone concave area showed a low genetic correlation estimate and a combination of these traits in a weighted index may give a better prediction of keel bone damage than each of the traits on their own. Tibiotarsal bone radiopacity had significant positive genetic correlation estimates with humerus bone, body weight, and egg traits [16]. Radiopacity of tibiotarsal and keel bone have been shown to have a favourable genetic relationship when measured before the onset of experimentally induced keel bone fractures [47]. Tibiotarsal bone radiopacity can, therefore, be a useful indicator trait to improve the general skeleton while at the same time taking account of body weight and egg production. The methods to automate keel bone phenotyping developed herein can be adapted to automate the tibiotarsal bone radiopacity phenotype proposed by Wilson et al. [32]. This would allow poultry breeders to compute phenotypes for both keel bone and tibiotarsal bone from the same radiographic images.

### Relationship between keel bone deviations and fractures

For keel bone fractures and deviations, our estimate of the genetic correlation (0.70) was approximately twice the estimate of the phenotypic correlation (0.31) and nearly four times the residual correlation (0.17). This suggests that the two traits share many genetic factors, while their environmental causes are more distinct. For breeding companies, this strong genetic correlation offers an opportunity to improve both keel deviations and fractures simultaneously through genetic selection. However, since their environmental drivers likely differ, as also suggested by previous studies, management practices may need to be tailored to each trait. For example, perch design may influence keel bone deviations [47–50], whereas fractures are more often linked to external trauma from collisions [51, 52] in combination with internal pressure [23]. Pathological findings support this distinction, with deviations occurring near the middle of the keel and fractures toward the end [5, 31].

### Relationships between keel bone traits and pelvic capacity

The pelvic cavity and keel bone are anatomically adjacent to each other. The pathological findings in previous studies attribute fractures to internal pressure exerted by the pelvic cavity contents on the keel bone [23]. Our findings suggest that a larger pelvic capacity is associated with a smaller keel concave area (−0.41 (−0.54) phenotypic (genetic) correlation; Table 1), and the concave area itself has inverse relationship with keel deviations and fractures. It is still unclear which component of the pelvic cavity is linked to an increased risk of keel bone fractures. It could be the result of a bird having a relatively large egg size in relation to its pelvic cavity size, or of eggs laid by early matured birds with a relatively small pelvic cavity [5, 9, 16]. Combining data of age-at-first-egg, egg size or weight, and keel bone concave area could help better understand the interplay between keel bone damage and pelvic cavity contents.

## Conclusion

In this study, automated methods for measuring keel bone radiography images were developed and evaluated for their genetic correlations with keel bone fractures and deviations. The keel bone concave area was found to be a reliable and heritable phenotype of keel bone that breeders can use for genetic selection, possibly in combination with tibiotarsal bone radiopacity, to reduce keel bone deviations and fractures in laying hens. The methods developed can also be adapted to measure other bones (e.g., tibiotarsla) and objects (e.g., eggs), allowing breeders to quickly compute phenotypes for keel bone, tibiotarsal bone, and egg size from the same radiography. The developed methods are also well-suited for large-scale studies to evaluate different housing environments and nutrition strategies aimed at improving keel bone conditions.

## Supplementary Information

Below is the link to the electronic supplementary material.

Supplementary Material 1

Supplementary Material 2

Supplementary Material 3

## Data Availability

The data analysed during the current study are not available for publication at the moment due to the needs of the current project.
