# Supplementary material for: Genetics of digital phenotypes of keel bone in layer chickens and correlations with keel bone fractures and deviations
Source: Genet Sel Evol. 2025 Nov 27;57:69. doi: 10.1186/s12711-025-01016-7 (PMC12661725; doi:10.1186/s12711-025-01016-7)
Supplement: Supplementary file 1 — Supplementary Material 1 [file 12711_2025_1016_MOESM1_ESM.docx]

**Table S2 Estimates of heritability ± standard error using single-trait GREML, including both Bovans Brown and Lohmann Brown birds (full data) or only Bovans Brown birds (partial data)**

|  | **Heritability ± standard error** | |
| --- | --- | --- |
|  | **Full data** | **Partial data** |
| Keel bone deviations size^a^ | 0.27±0.09 | 0.26±0.11 |
| Keel bone fractures count^a^ | 0.27±0.09 | 0.25±0.10 |
| Keel bone callus size^a^ | 0.39±0.09 | 0.35±0.10 |
| Pelvic capacity^b^ | 0.32±0.09 | 0.25±0.10 |
| Culling body weight | 0.44±0.09 | 0.41±0.11 |
| Keel bone concave area^c^ | 0.36±0.09 | 0.35±0.10 |
| Ratio of keel bone length to mid-depth^c^ | 0.05±0.09 | 0.07±0.11 |
| Whole keel bone radiopacity^c^ | 0.001±0.00 | 0.02±0.11 |
| Tibiotarsal radiopacity^d^ | 0.52±0.08 | 0.51±0.09 |
| Keel bone cranial fifth radiopacity^c^ | 0.003±0.00 | 0.000±0.00 |
| ^a^ scoring dissected keel bones by nine operators, then adjusting scores for the operator effect  ^b^ requires human-operator to measure pelvic width and depth by digital caliper  ^c^ fully automated measurement on the radiographs of chicken whole-body  ^d^ requires human-operator to indicate key points on radiographs of dissected tibiotarsal bones, from these points computes the tibiotarsal mid-shaft radiopacity | | |
