# Supplementary figures and images for: Genetics of digital phenotypes of keel bone in layer chickens and correlations with keel bone fractures and deviations
Source: Genet Sel Evol. 2025 Nov 27;57:69. doi: 10.1186/s12711-025-01016-7 (PMC12661725; doi:10.1186/s12711-025-01016-7)

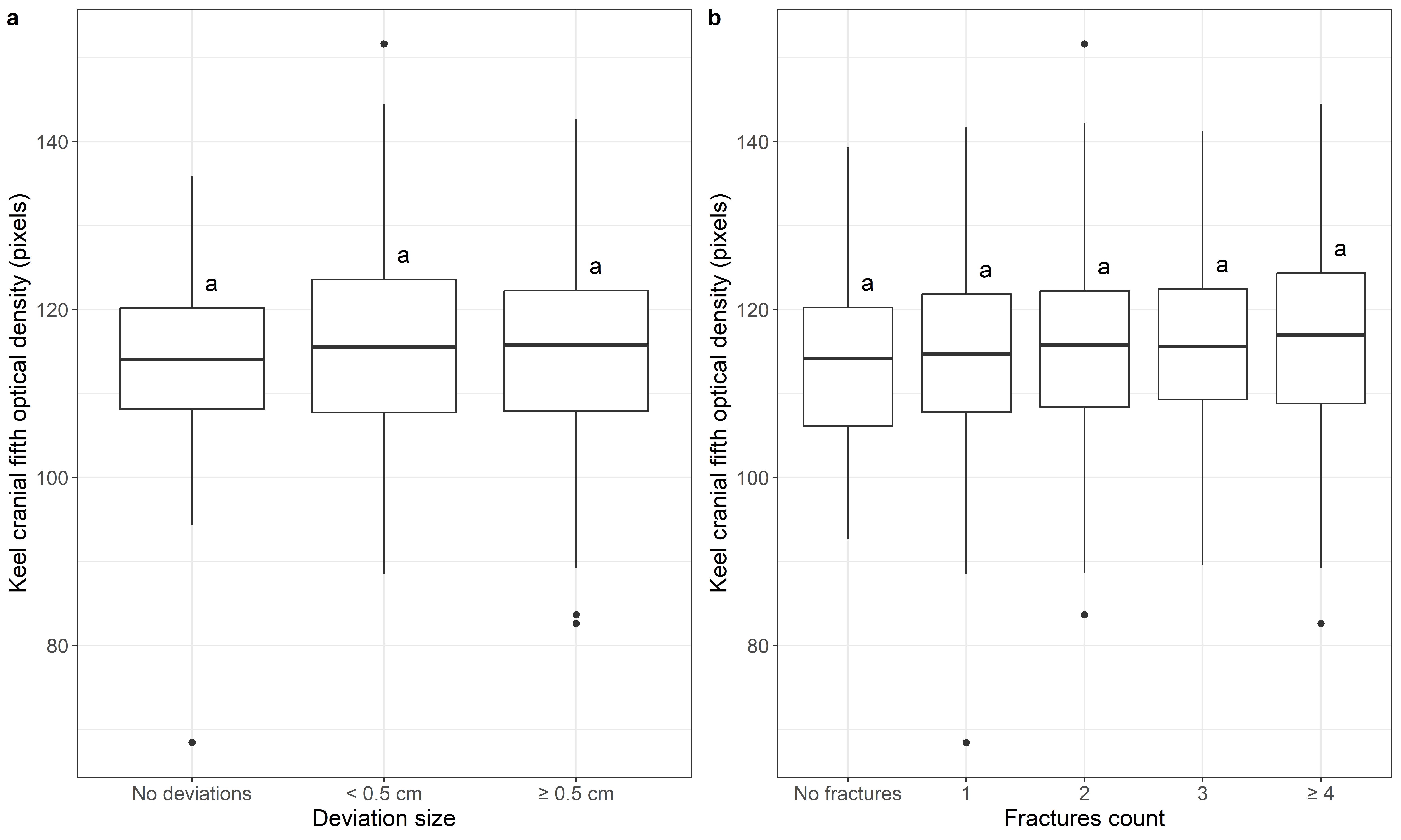

Supplement: Supplementary file 2 — Supplementary Material 2 [file 12711_2025_1016_MOESM2_ESM.jpeg]
