# Supplementary material for: Genetics of digital phenotypes of keel bone in layer chickens and correlations with keel bone fractures and deviations
Source: Genet Sel Evol. 2025 Nov 27;57:69. doi: 10.1186/s12711-025-01016-7 (PMC12661725; doi:10.1186/s12711-025-01016-7)
Supplement: Supplementary file 3 — Supplementary Material 3 [file 12711_2025_1016_MOESM3_ESM.docx]

**Table S1 Estimates ± standard error of heritability (diagonals), genetic correlation (lower off-diagonals) and phenotypic correlation (upper off-diagonals) of traits evaluated on Bovans Brown birds**

|  | **Keel bone deviations**  **size^a^** | **Keel bone fractures**  **count^a^** | **Pelvic capacity^b^** | **Culling body**  **weight** | **Keel bone concave**  **area^c^** | **Ratio of keel bone length to**  **mid-depth^c^** | **Whole keel bone**  **radiopacity^c^** | **Tibiotarsal bone radiopacity^d^** | **Keel bone cranial**  **fifth radiopacity^c^** |
| --- | --- | --- | --- | --- | --- | --- | --- | --- | --- |
| Keel bone deviations size | **0.29±0.09** | 0.32±0.04 | 0.11±0.04 | -0.01±0.04 | -0.29±0.04 | 0.35±0.03 | 0.23±0.03 | -0.14±0.04 | 0.08±0.03 |
| Keel bone fractures count | 0.66±0.22 | **0.29±0.06** | 0.08±0.04 | -0.04±0.04 | -0.39±0.03 | 0.09±0.04 | 0.19±0.04 | -0.14±0.04 | 0.06±0.04 |
| Pelvic capacity | -0.03±0.32 | -0.17±0.26 | **0.27±0.06** | 0.51±0.03 | -0.38±0.04 | 0.01±0.04 | 0.01±0.04 | 0.08±0.04 | -0.04±0.04 |
| Culling body weight | -0.19±0.25 | 0.02±0.18 | 0.53±0.18 | **0.42±0.09** | -0.11±0.04 | 0.02±0.04 | 0.09±0.04 | 0.33±0.04 | -0.10±0.04 |
| Keel bone concave area | -0.64±0.21 | -0.53±0.15 | -0.42±0.20 | -0.29±0.20 | **0.38±0.08** | -0.06±0.04 | -0.32±0.03 | 0.10±0.04 | -0.14±0.04 |
| Ratio of keel bone length to mid-depth | 0.66±0.48 | 0.72±0.42 | -0.34±0.58 | -0.26±0.45 | -0.34±0.40 | **0.13±0.06** | 0.27±0.03 | -0.04±0.04 | 0.10±0.04 |
| Whole keel bone radiopacity | 0.82±0.24 | 0.68±0.24 | -0.22±0.28 | -0.06±0.28 | -0.76±0.20 | 0.57±0.60 | **0.09±0.03** | -0.11±0.04 | 0.88±0.09 |
| Tibiotarsal bone radiopacity | -0.36±0.21 | -0.33±0.15 | 0.12±0.21 | 0.28±0.16 | 0.22±0.17 | 0.04±0.35 | -0.42±0.24 | **0.52±0.09** | -0.10±0.04 |
| Keel bone cranial fifth radiopacity | 0.44±0.50 | 0.20±0.54 | -0.35±0.53 | 0.09±0.51 | -0.43±0.44 | 0.18±0.80 | 0.77±0.50 | -0.39±0.45 | **0.05±0.03** |
| ^a^ scoring keel bones post-dissection by nine operators, then adjusting the scores for the operator effect  ^b^ requires human-operator to measure pelvic width and depth by digital calliper  ^c^ fully automated measurement on the radiographs of chicken whole-body  ^d^ requires human-operator to indicate key points on radiographs of the dissected tibiotarsal bones, from these points computes the tibiotarsal bone mid-shaft radiopacity | | | | | | | | | |
